# The skin microbiota of preterm infants and impact of diaper change frequency

**DOI:** 10.1371/journal.pone.0306333

**Published:** 2024-08-01

**Authors:** Noelle E. Younge, D. Joshua Parris, Daniel Hatch, Angel Barnes, Debra H. Brandon

**Affiliations:** 1 Department of Pediatrics, Duke University School of Medicine, Durham, NC, United States of America; 2 Kimberly-Clark Corporation, Neenah, WI, United States of America; 3 Duke University School of Nursing, Durham, NC, United States of America; RCSI & UCD Malaysia Campus (formerly Penang Medical College), MALAYSIA

## Abstract

**Objective:**

To evaluate the impact of diaper change frequency, clinical characteristics, and skin health metrics on development of the skin microbiota in preterm infants.

**Design:**

A randomized controlled parallel design was used.

**Methods:**

Medically stable preterm infants born <33 weeks’ gestation were randomized to receive diaper changes at a frequency of every 3-hours or every 6-hours. Skin swabs were collected longitudinally from the diapered skin (buttocks) and chest. Skin pH and transepidermal water loss were measured with each sample collection. Stool samples were collected from the diaper. The microbiome at each site was characterized by 16S rRNA gene sequencing. Associations between microbiome features, diaper change frequency, and other covariates were examined using mixed effect models and redundancy analysis.

**Results:**

A total of 1179 samples were collected from 46 preterm infants, beginning at a median postnatal age of 44 days and continuing through hospital discharge. Alpha-diversity of the skin microbiota increased over time, but did not differ significantly between 3-hour (n = 20) and 6-hour (n = 26) diaper change groups. Alpha-diversity of the skin microbiota was inversely correlated with skin pH, but not transepidermal water loss. Microbiota community structure differed significantly between body sites (buttocks, chest, and stool) and between individuals. Among samples collected from the diapered skin, diaper change frequency, infant diet, antibiotic exposure, and delivery mode accounted for minor proportions of the variation in microbiota community structure between samples. Relative abundances of multiple genera differed between 3- and 6-hour diaper change groups over time.

**Discussion/Conclusion:**

The diversity and composition of the diapered skin microbiota is dynamic over time and differs from other body sites. Multiple factors including interindividual effects, diaper change frequency, diet, and antibiotics contribute to variation in the diapered skin microbiota.

## Introduction

The microbiota plays important roles in infant health and development, including maturation of the immune system, intestinal physiology, and protection against infection [1–4]. The microbiota of preterm infants is altered relative to healthy full-term infants. For example, the skin and fecal microbiotas of preterm infants are characterized by dominance of hospital-associated facultative anaerobes (e.g., *Staphylococcus*, *Klebsiella*, *Enterococcus*), while commensal organisms characteristic of the full-term infant intestinal microbiota (e.g., *Bifidobacterium*) are lacking [5–8]. Enrichment of pathogenic organisms in the intestinal microbiota of preterm infants is thought to contribute to the pathogenesis of neonatal morbidities including necrotizing enterocolitis and late-onset sepsis [9,10]. Numerous studies have identified associations between various neonatal exposures and treatments with fecal microbiota profiles in preterm infants, but the development of the skin microbiota and impact of skin care practices have been less well-characterized.

In addition to effects on the developing microbiota, environmental exposures in the neonatal intensive care unit (NICU) may adversely impact other areas of infant development. For example, exposures to light, noise, painful procedures, and even routine caregiving events such as diaper changing may be experienced by the infant as uncomfortable, stress-provoking, and/or disruptive to sleep. Exposure to chronic stress and pain in the NICU has been associated with poor habituation and stress responses, altered brain development, and adverse neurodevelopmental outcomes [11–16]. In effort to reduce the potential negative impacts of the NICU environment on infant development, developmental care programs have been widely adopted in NICUs around the world. Bundled care, in which multiple care activities are grouped into a single caregiving event, is one developmental care strategy that is commonly implemented to reduce infant stress in the NICU [17,18]. However, the inclusion and exclusion of specific care activities during bundled care have not been systematically studied.

We conducted a randomized, controlled trial to study the impact of every 3-hour (unit standard of care) vs. every 6-hour diaper change frequency on infant vital sign stability and skin health in medically stable, convalescing preterm infants receiving bundled care [19]. We found that bundled care events that included diaper changes were more often associated with vital sign changes (e.g. increased heart rate) than bundled care events without a diaper change. Measures of skin health, including skin pH, transepidermal water loss (TEWL), and Neonatal Skin Condition Scores (NSCS), were not significantly different between infants who received 3- or 6-hour bundled diaper care. These findings suggest that reducing diaper change frequency may be one approach to reduce caregiving stress in the NICU without negative effects on skin health, but the impact on the development of the skin microbiota is not known. Factors such as decreased frequency of skin cleansing and exposure to air may alter the skin environment and its selective pressures on the developing skin microbiota.

Here, we report the analysis of the skin microbiota of preterm infants enrolled in the bundled care trial. The objectives of the study were to evaluate the impact of diaper change frequency and other clinical factors on the development of the skin microbiota. Second, we sought to explore the relationship between the skin microbiota, stool microbiota and skin health measures. We hypothesized that the diapered skin microbiome would be influenced by the fecal microbiome.

## Materials and methods

### Clinical trial

The details of this randomized controlled parallel trial of bundled diaper care on infant vital signs and skin health have been published [19]. While planned from the beginning of the study, the microbiome outcome was added to clinical trial registry retrospectively because the specific variable to be analyzed was not confirmed at the establishment of the registry (see Clinicaltrials.gov registry # NCT03370757) and CONSORT reporting guidelines (S1 Table) were used [20]. Human subjects’ approval was obtained from the Duke University Health System Institutional Review Board and written informed consent was obtained prior to any data collection; infants were recruited between December 2018 and July 2019. In brief, preterm infants born <33 weeks’ gestation without neonatal abstinence syndrome, or a pre-existing genetic skin condition were eligible for inclusion in the study. The intervention was initiated when the infants were medically stable and tolerating enteral feedings on an every 3-hour schedule in the Neonatal Intensive Care Unit. Due to the nature of the intervention study staff and nurses who implemented the intervention could not be blinded to group assignment. Study staff had to be aware of group assignment to schedule and collect data. Infants were stratified by birth weight (≤800 grams, >800 to 1150 grams, >1150 grams) and randomized to receive either 3- or 6- hour diaper changes. Infants in both groups received standard bundled care every 3-hours for all other care activities (e.g., feeding, temperature check) as dictated by the infant’s care needs. If an infant in the 6-hour diaper change group was noted to have a soiled diaper (stool present) with the diaper in place during a 3-hour caregiving interval, the diaper was changed regardless of whether they were due for a diaper change and the timing of the next diaper change was scheduled for 6-hours from that bundled care event. Forty-six infants completed the study. The study was powered for the skin health outcomes of transepidermal water loss.[19] Although it was not possible to accurately predict the effect size of diaper change frequency on the skin microbiome of infants *a priori*, this study is powered more robustly than previous studies of infant cutaneous microbiomes which have shown significant differences in alpha and beta diversity metrics due to emollient use and the presence/absence [21,22].

### Sample and clinical data collection

Skin swabs and fecal samples were collected from infants up to 3 times per week from the time of initiation of the intervention until discharge. Nova Biostorage FLOQ swabs with cuvette were used to collect samples from the buttocks as well as the chest as a control site outside of the diapered area. Chest samples were obtained first by swabbing the chest area two back and forth strokes on each covering approximately 3 centimeters per stroke. Skin buttocks samples were obtained prior to standard cleansing during the diaper change. If stool was present in the diaper at the time of the sampling, the diaper was used to remove as much stool as possible from the buttocks before proceeding with sample collection. The left and right buttocks were swabbed with two back and forth strokes on each buttock for a total of four strokes covering approximately 3 centimeters per stroke. Stool samples were retrieved from the diaper. All samples were placed in the -20 freezer in the neonatal unit immediately after data collection. Samples were then transferred to the -80 freezer within 48 hours of collection.

Skin pH and TEWL were measured at each skin site at the time of sample collection. Skin pH was measured using the ExStik^TM^ PH100 meter by Extech^®^ instruments. The meter contains a flat surface electrode that was placed at the center of the test site (chest or buttocks). TEWL values were obtained using the DermaLab^®^ TEWL probe (Cortex Technology, Hadsund, Denmark), which consists of an open probe with paired sensors at different distances from the skin. Humidity and temperature are measured in each sensor to calculate vapor pressure gradients and the difference between gradients is representative of TEWL at that point on the skin [23].

Clinical and demographic data were collected from the electronic health record. Time of sample collection was recorded as days since randomization to reflect the duration of intervention, as well as the infant’s chronological age in days and the infant’s postmenstrual age (PMA; defined as birth gestational age plus chronological age) at sampling. Metadata collected for each sample included skin pH, TEWL, and NSCS; presence of diaper rash at time of sampling; skin care treatments at sampling (e.g., barrier cream); current and prior antibiotic exposures; and infant diet (maternal human milk, donor human milk, and/or formula).

### Sample processing

Genomic DNA was isolated from stool and skin swabs using the PowerSoil® DNA Isolation Kit (Qiagen) following the manufacturer’s instructions, with the exception that the complete swab head (skin swabs) or approximately 250 mg of stool was used as an alternative to the recommended 250 mg of soil for cell lysis. The purified DNA was stored at -20°C until PCR amplification. Primers targeting the 16S rRNA gene (341F CCTACGGGNGGCWGCAG/ 785R GACTACHVGGGTATCTAATCC) were used for PCR with methods via the bacterial tag-encoded flexible-Titanium (FLX) amplicon pyrosequencing (bTEFAP^®^) DNA analysis service (MR DNA, Shallowater, TX) [24] Each sample underwent a single-step 35 cycle PCR using HotStarTaq Plus Master Mix Kit (Qiagen, Valencia, CA) under the following conditions: 95°C for 5 minutes, followed by 30 cycles of 95°C for 30 seconds; 53°C for 40 seconds and 72°C for 1 minute; after which a final elongation step at 72°C for 10 minutes was performed. Negative controls of blank swab extractions and no-template controls for each set of PCR reactions were used to verify extraction yield and lack of contamination during PCR setup. Following PCR, all amplicon products were mixed in equal concentrations and purified using calibrated solid phase reverse immobilization beads. Samples were sequenced utilizing the Illumina MiSeq chemistry following manufacturer’s protocols. Sequencing data were processed in QIIME2 using the DADA2 pipeline with default parameters.

### Analysis

Demographic and clinical data were presented using median and interquartile ranges for continuous variables and counts and proportions for categorical data. Comparisons between the 3- and 6-hour bundled care groups were performed using Wilcoxon rank-sum tests for continuous variables and Fisher’s exact test for categorical variables.

Alpha-diversity indices were calculated using QIIME2 [25]. Linear mixed effects models were used to evaluate the relationships between microbiota diversity, as measured by the Shannon Diversity Index (outcome variable) with bundled diaper care group (3-hour vs. 6-hour) and time (predictor variables), using the lme4 R package. We made no assumptions about how the residuals at each time point would correlate with each other, therefore we used an unstructured covariance matrix in the linear mixed effects models. The time variable was assessed as days since randomization. An interaction between time and bundled care group was included in the model in order to assess whether changes in diversity over time varied by bundled care group. Additional covariates considered as fixed effects in the models were PMA and chronological age in weeks at randomization (centered), use of diaper skin care products at sampling, receipt of antibiotics at sampling, and infant diet (maternal or donor human milk, formula, mixture human milk and formula, or nil per os) at sampling. Individual subject was included as a random effect in the models to account for correlation of repeated measures within individuals. A second set of models was then constructed for each site (buttocks, chest, and stool) in which only bundled care group, time since randomization, and any additional covariates with a p value of ≤0.10 in the full model were included. Associations between microbiota diversity and skin pH and TEWL were explored using Pearson correlation.

Redundancy analysis (RDA) was used to model the relationship between sample metadata and microbiota composition using the vegan package in R (version 4.1.0). Variables considered in the model included site (buttocks, chest, or stool), bundled care group, time on study (week), delivery mode (vaginal or cesarean delivery), subject, number of antibiotic courses, PMA, and diet (human milk). To specifically evaluate the effect of the bundled care intervention on the microbiota of the diapered skin, a separate model was constructed that included only samples collected from the buttocks. To reduce zero values from the analysis, taxonomy was collapsed to the genera level and only genera with an average total abundance greater than 0.1% were included. Relative abundance data were centered log ratio transformed using the clr function in R (version 4.1.0) prior to running the model. Statistical significance of the model and individual factors was assessed using the Kruskal-Wallis test with post-hoc pairwise tests as indicated using the vegan package in R (version 4.1.0).

The R package MaAsLin2 (version 1.8.0) was used to examine associations between bacterial genera and clinical features. Multivariable models were run separately for each site (buttocks, chest, and stool), with bundled diaper care group (6-hr group vs. 3-hr reference group), time (days since study initiation), interaction between time and group, delivery mode, human milk diet, PMA at study initiation, presence of stool in the diaper prior to sampling (for skin buttocks only), and current antibiotics included as fixed effects and individual subject included as a random effect. Default parameters were used in the model with the exception that the minimum abundance threshold was set as 0.0001.

## Results

**Fig 1:**
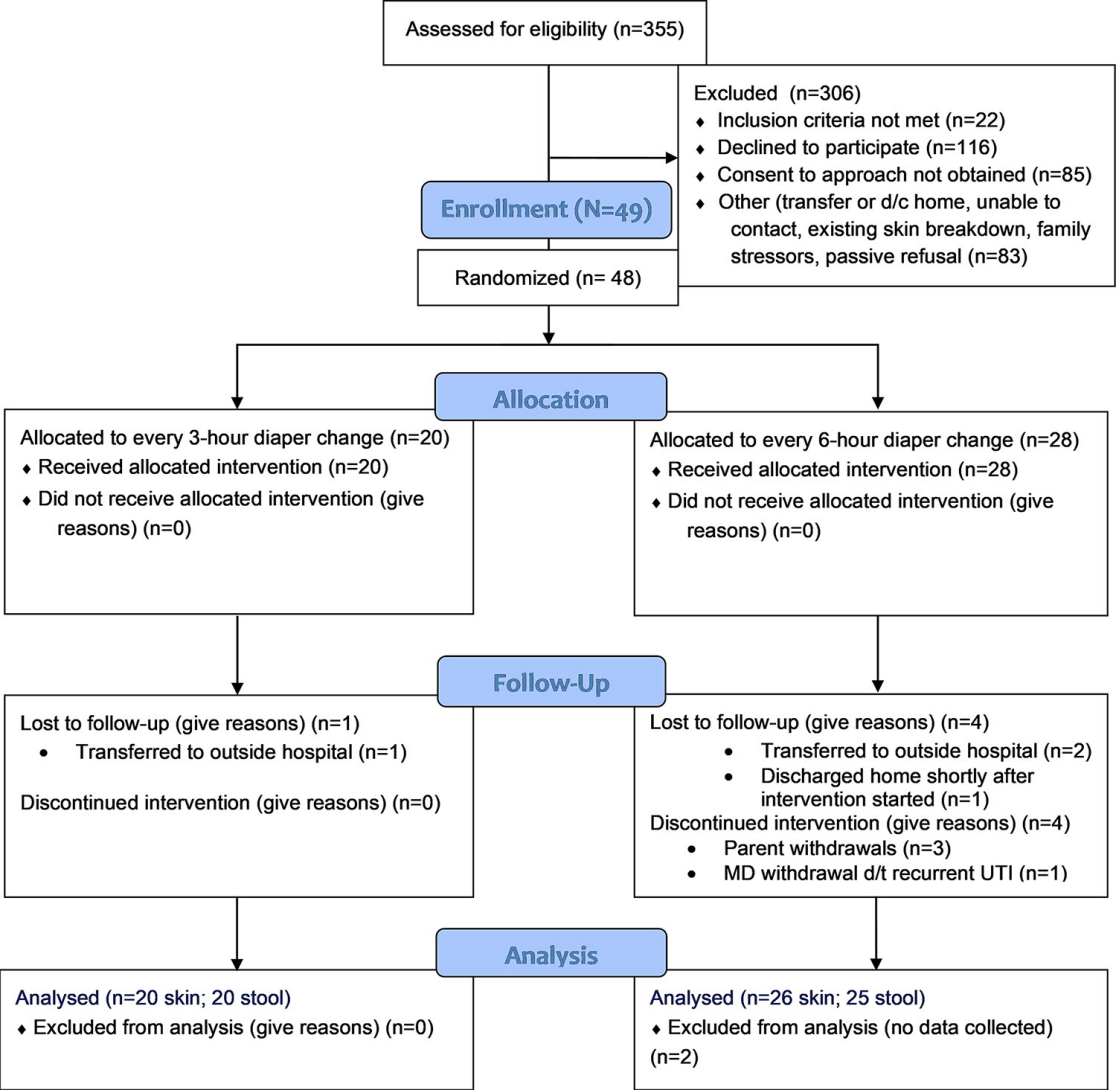
Consort flow diagram. Forty-six infants completed the study and were included in data analyses.

Among 46 infants enrolled in the trial, the median gestational age at birth was 28 weeks and the median PMA at the initiation of the intervention was 34.6 weeks (Fig 1 and Table 1). Samples were collected beginning at a median postnatal age of 44 days and continued for 6 weeks or until hospital discharge, whichever came first. Twenty infants were randomized to the 3-hour bundled care group and 26 infants to the 6-hour bundled care group. All infants in both groups had a history of antibiotic exposure prior to the initiation of intervention. No infants had culture-positive sepsis, but a minority of infants in both groups had urinary tract infections. Two infants (n = 1 per group) had a history of abdominal surgery for necrotizing enterocolitis and/or intestinal perforation. Most infants received a combination of human milk and infant formula during the intervention period.

**Table 1:** Infant characteristics.

	3-hour Bundled Diaper Care N = 20	6-hour Bundled Diaper Care N = 26	P
Birth gestational age, weeks	27.5 (26–29)	29 (26–31)	0.35
Antenatal antibiotics, n (%)	10 (53)	13 (52)	>0.99
Cesarean delivery, n (%)	12 (60)	15 (58)	>0.99
Male sex, n (%)	13 (65)	8 (31)	0.04
Race, n (%)			0.84
White	8 (40)	13 (50)	
Black or African American	6 (30)	8 (31)	
Asian	1 (5)	1 (4)	
Other or more than one race	5 (25)	4 (15)	
Hispanic or Latino ethnicity, n (%)	3 (15)	2 (8)	0.64
Diet*, n (%)			
Maternal breast milk	15 (75)	19 (73)	>0.99
Donor human milk	8 (40)	14 (54)	0.40
Infant formula	17 (85)	16 (62)	0.11
Total antibiotic courses, n (%)	3.5 (2–6)	3 (2–6)	0.45
Surgical necrotizing enterocolitis and/or intestinal perforation, n (%)	1 (5)	1 (4)	>0.99
Urinary tract infection, n (%)	4 (20)	3 (12)	0.68

*Defined as any receipt of maternal breast milk, donor human milk, or infant formula during the intervention period.

A total of 1179 samples (528 skin swabs from buttocks, 520 skin swabs from chest, and 131 stool samples) were sequenced for microbiota analysis with sequence counts ranging from 9,209 to 109,360 and a mean (± SD) of 30,980 ± 11,264 sequences per sample. This sequencing depth was sufficient to capture alpha-diversity within samples (S1 Fig). This included a mean of 35,445 sequences per sample for buttock swabs, 27,553 sequences for chest swabs, and 26,585 sequences for stool samples. Raw sequence data is publicly available in the NCBI SRA archive and accessible under Bioproject ID PRJNA1015700 (http://www.ncbi.nlm.nih.gov/bioproject/PRJNA1015700).

**Fig 2:**
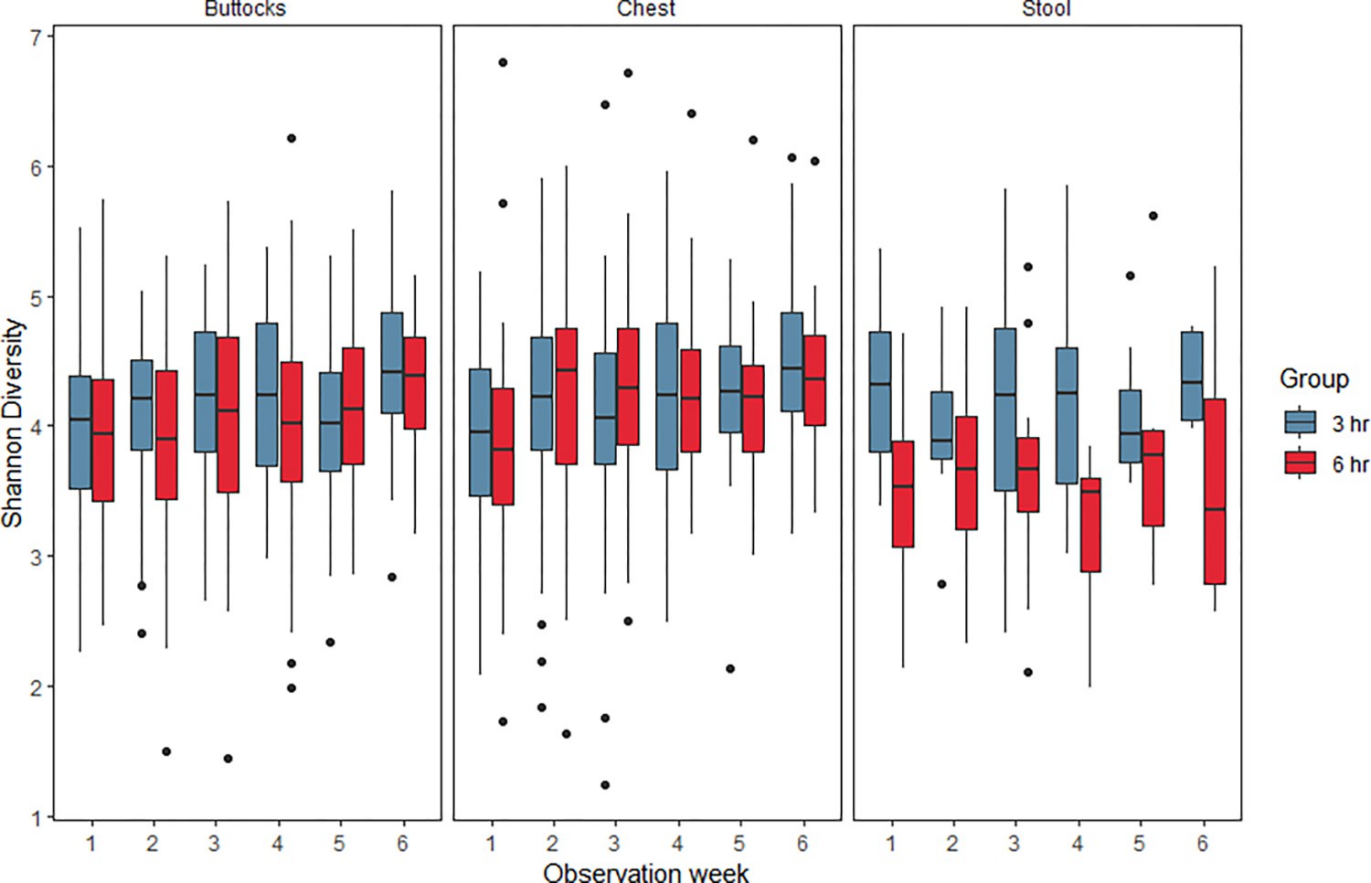
Diversity of the microbiota. Alpha-diversity of the microbiota, as measured by the Shannon Diversity Index, is presented by site (skin buttocks, skin chest, and stool), observation week (weeks since initiation of intervention), and bundled care group (every 3-hour vs. every 6-hour diaper changes in blue and red, respectively). Diversity of the microbiota increased over time in all sites. Diversity was higher in the stool microbiota of infants treated with every 6-hour diaper changes than every 3-hour diaper changes, but there were no significant time*group interactions at any of the sites.

The alpha-diversity of the microbiota was higher among skin samples collected from the buttocks and chest than the stool (p<0.001 for chest vs. stool and p = 0.004 for buttocks vs. stool by pairwise Wilcoxon rank-sum tests with Benjamini-Hochberg correction). Linear mixed effects models were used to evaluate the relationships between alpha-diversity of the microbiota, bundled care group, time, and other covariates. Microbiota diversity of the skin at both the buttocks and chest sites increased over time in both groups, but bundled care group, the interaction between bundled care group and time, and other clinical covariates were not significant in the models (Fig 2 and Table 2). Diversity of the stool microbiota was lower in the 6-hour bundled care group but the interaction between group and time was not significant.

**Table 2:** Linear mixed effects models of shannon diversity of the microbiota by body site.

	Skin-Buttocks		Skin-Chest		Stool	
Variable	Model 1* β [95% CI]	Model 2* β [95% CI]	Model 1* β [95% CI]	Model 2* β [95% CI]	Model 1* β [95% CI]	Model 2* β [95% CI]
Intercept	**4.21** ** **[3.85, 4.57]**	**4.03** ** **[3.79, 4.26]**	**3.89** ** **[3.51, 4.26]**	**3.92** ** **[3.73, 4.11]**	**4.10** ** **[3.54, 4.66]**	**3.96** ** **[3.65, 4.27]**
BC Group	-0.10 [-0.43, 0.22]	-0.19 [-0.47, 0.09]	0.12 [-0.17, 0.41]	0.14 [-0.08, 0.36]	-0.34 [-0.79, 0.11]	**-0.46** ** **[-0.83, 0.09]**
Time (days)	**0.01** ** **[0.00, 0.01]**	0.00^†^ [-0.00, 0.01]	**0.01** ** **[0.00, 0.02]**	**0.01** ** **[0.00, 0.01]**	**0.01** ** **[0.00, 0.02]**	**0.01** ** **[0.00, 0.01]**
PMA at randomization (weeks)	0.01 [-0.09, 0.10]		0.05 [-0.03, 0.12]		0.06 [-0.07, 0.19]	
Age at randomization (weeks)	-0.01 [-0.07, 0.05]		-0.03 [-0.08, 0.02]		-0.03 [-0.11, 0.05]	
Use of skin product(s)	-0.00 [-0.11, 0.10]		0.12^†^ [-0.02, 0.26]	0.12^†^ [-0.02, 0.25]	0.12 [-0.09, 0.33]	
Antibiotics	-0.10 [-0.29, 0.10]		-0.01 [-0.25, 0.24]		-0.01 [-0.38, 0.35]	
Diet at sampling						
Human milk	-0.25^†^ [-0.52, 0.03]	-0.23^†^ [-0.48, 0.02]	-0.05 [-0.36, 0.27]		-0.31 [-0.77, 0.15]	
Formula	0.11 [-0.16, 0.38]	0.11 [-0.14, 0.36]	-0.01 [-0.33, 0.31]		0.01 [-0.49, 0.52]	
Mix	-0.26^†^ [-0.55, 0.03]	-0.22 [-0.49, 0.05]	0.13 [-0.21, 0.46]		-0.31 [-0.79, 0.17]	
BC Group* Time	-0.01 [-0.02, 0.00]		-0.00 [-0.01, 0.01]		-0.01 [-0.02, 0.01]	

*Model 1 includes all co-variates. Model 2 includes bundled care group, time, and co-variates with p≤0.10 in Model 1.

**p<0.05.

^†^p≤0.10.

BC, Bundled care, PMA, Postmenstrual age.

Next, RDA was used to evaluate factors associated with variation in microbiota community structure between samples. First, samples from all sites were included in an RDA model. The resulting model explained 49.8% of the variation in microbiota community structure between samples (p<0.001, S2 Fig and S2 Table). Of all variables considered in the model, site (buttocks, chest, or stool) accounted for most of the variation between samples (30.0%, p<0.001), followed by individual subject (6.1%, p<0.001), and delivery mode (3.3%, p<0.001). Bundled care group (3-hour vs. 6-hour) accounted for around 3% of variation (p<0.001). Taxonomic profiles grouped by site demonstrated that *Staphylococcus* and *Streptococcus* constituted nearly half of the community in chest swabs, while the composition of the buttocks skin microbiota was similar to the stool, with predominance of genera within the *Enterobacteriaceae* and *Clostridiaceae* families (S3 Fig).

**Fig 3:**
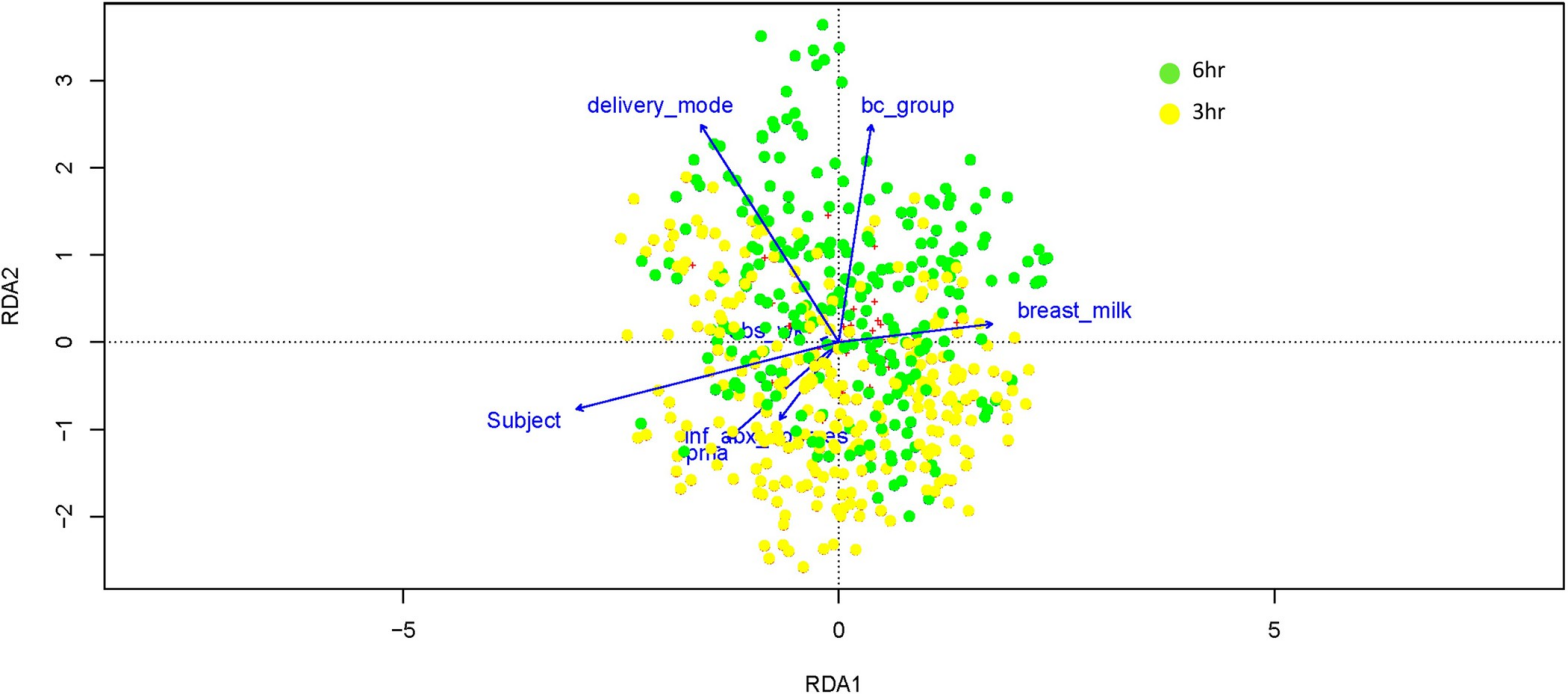
Community structure of the buttocks skin microbiota. Redundancy analysis (RDA) showing factors contributing to variation in microbiota composition between skin buttocks samples. The overall RDA model explained 27.9% of variance in microbiota composition. All factors considered in the model (diaper change frequency, number of antibiotic courses, human milk diet, observation week, delivery mode, postmenstrual age, and subject) contributed significantly to the model (p<0.001). Explanatory variables are represented by blue arrows. Samples are represented by circles and color-coded according to diaper change frequency (6-hour group in green, 3-hour group in yellow). Red crosses represent microbial taxa.

To further evaluate the effect of diaper change frequency and other factors on microbiota composition in the diapered skin, we constructed a separate RDA model that only included skin samples from the buttocks. The resulting model explained 27.9% of the variation in microbiota community structure between samples (Fig 3 and Table 3). Samples clustered within individual subjects, accounting for 7.4% of variation in the model (p<0.001; S4 Fig). Bundled diaper care group accounted for around 4% of overall variation (p<0.001). Other factors in the model including delivery mode and human milk diet also accounted for minor proportions of the variation in microbiota community structure.

**Table 3:** Redundancy analysis of diapered skin microbiota.

	Variance	F	Pr(>F)
Subject	7.39	21.39	<0.001
Delivery mode	5.29	15.32	<0.001
Bundled care group	4.33	12.54	<0.001
Postmenstrual age	3.90	11.29	<0.001
Total antibiotic courses	3.41	9.88	<0.001
Human milk	2.31	6.70	<0.001
Observation week	1.24	3.58	<0.001

**Fig 4:**
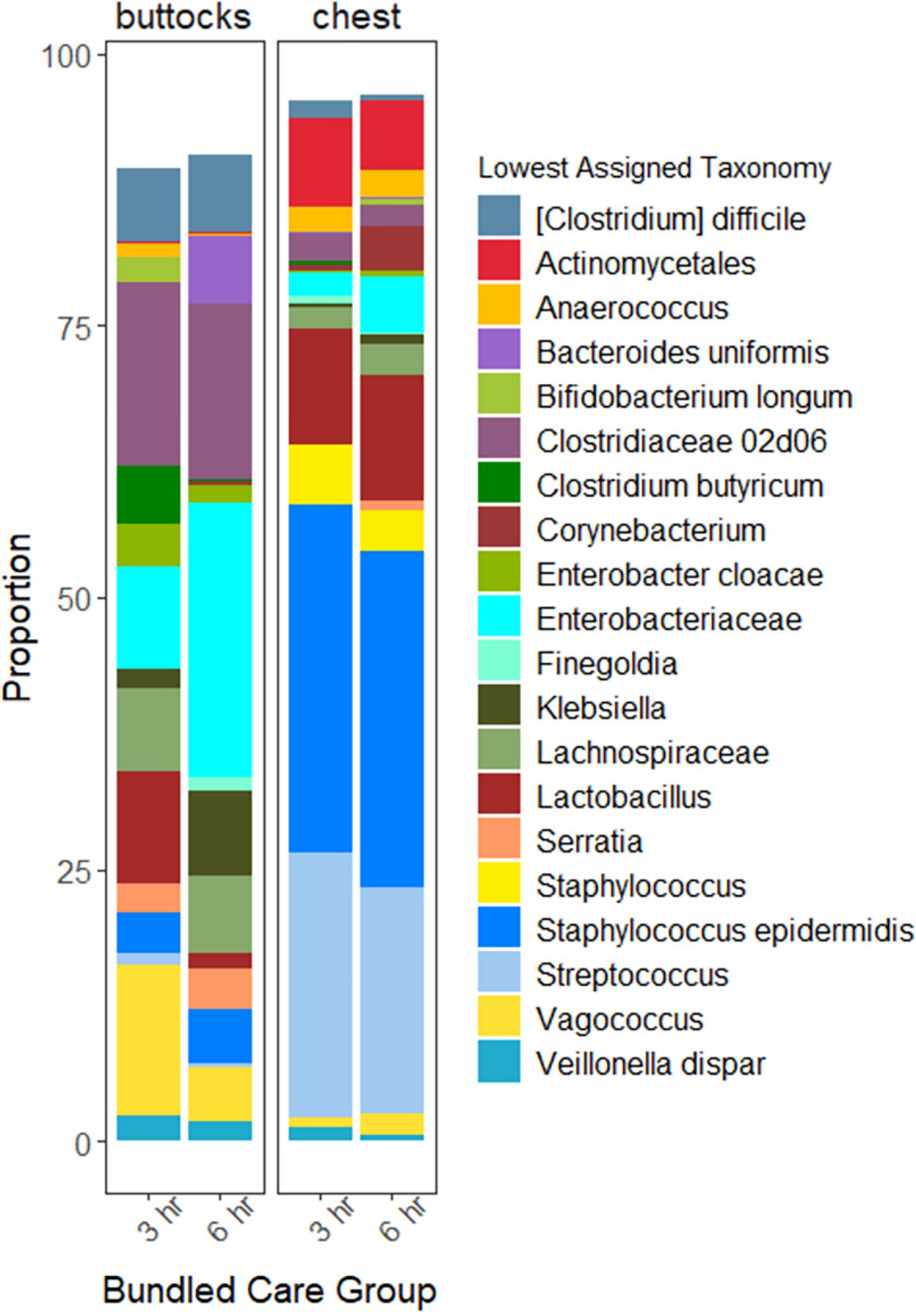
Skin microbiota profiles by site and diaper change frequency. Relative abundances of bacterial genera aggregated by site and bundled care group (every 3-hour vs. every 6-hour diaper change frequency). Gut-associated taxa including *Clostridium*, *Escherichia*, and *Klebsiella* had higher relative abundance in the skin buttocks, while *Staphylococcus* and *Streptococcus* had higher relative abundance in the skin chest.

**Fig 5:**
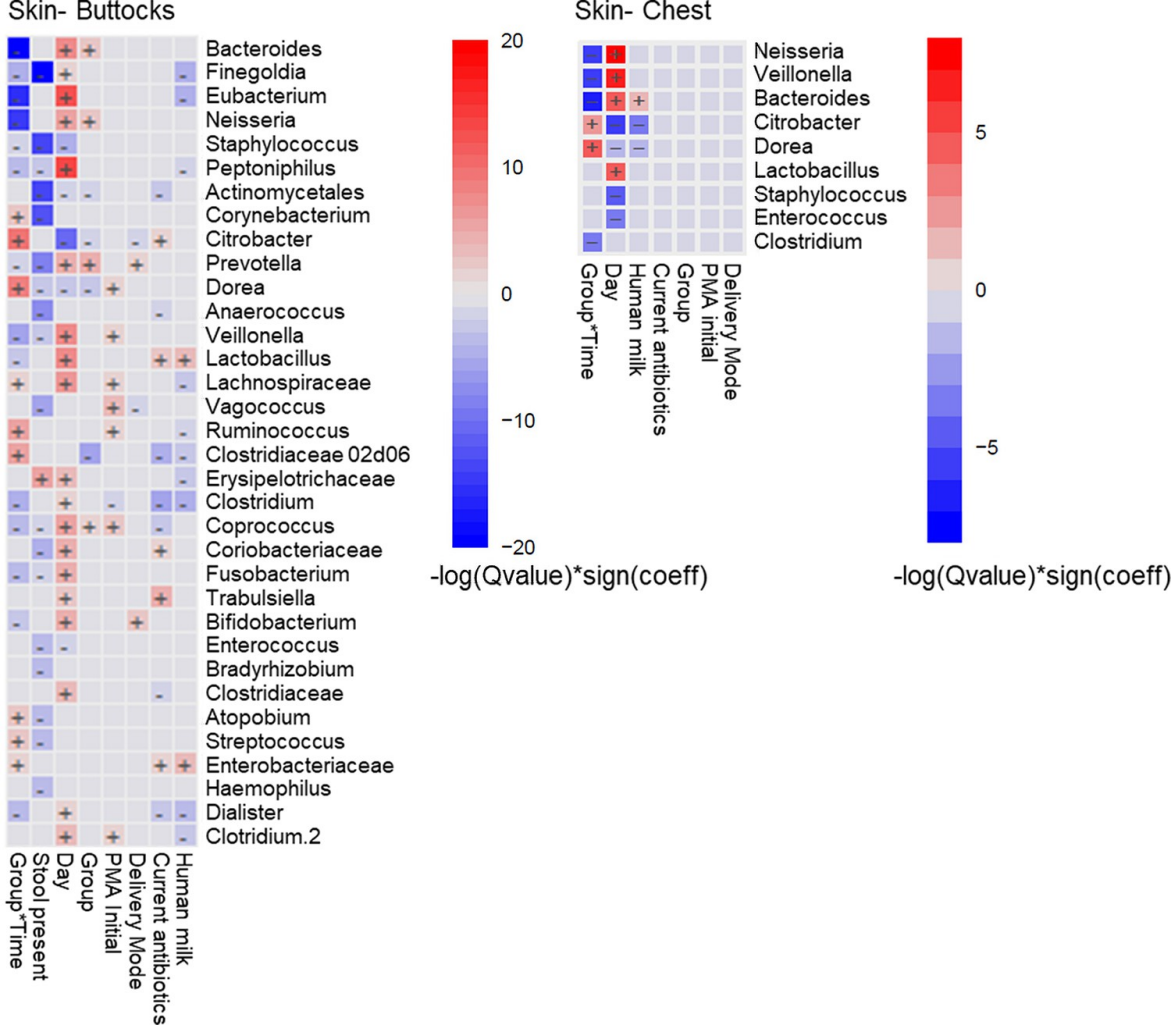
Association of diaper change frequency and clinical factors with relative abundances of bacterial Genera in the Skin Microbiota. Heatmap showing associations between relative abundances of bacterial genera (or lowest assigned taxonomic grouping) with diaper change group (6-hr vs 3-hr), time (days since study initiation), the interaction between group and time (Group*Time), human milk feeding, current antibiotic use, postmenstrual age (PMA) at study initiation, stool presence in diaper prior to sampling (for buttocks samples only), and delivery mode (vaginal vs. cesarean). Only genera with a significant association with at least one variable are shown (Q<0.05).

Taxonomic profiles of the skin microbiota among infants in the 3-hour and 6-hour bundled diaper care groups are presented in Fig 4. The relative abundances of bacterial genera were compared between groups and over time using mixed effects models for each site. In the buttocks samples, relative abundances of multiple genera varied over time (Fig 5). Genera that differed in relative abundance over time between groups (i.e., group x time interaction) included *Bacteroides*, *Eubacterium*, *Neisseria*, *Citrobacter*, *Dorea*, *Veillonella*, *Ruminococcus*, *Clostridiaceae 02d06*, and others. For some genera, the differences between groups over time were observed in both the diapered skin and chest microbiota (e.g., *Bacteroides*, *Neisseria*, *Dorea*, *Veillonella*), while other genera differed only in the diapered skin microbiota between groups (e.g., *Eubacterium*, *Citrobacter*, *Ruminococcus*, *Clostridiaceae* 02d06). In the diapered skin microbiota, the relative abundance of *Prevotella* was higher and the relative abundance of *Clostridiaceae 02d06* was lower in the 6-hour diaper change group. Receipt of human milk feedings was associated with higher relative abundance of *Enterobacteriaceae* and *Lactobacillus* but lower relative abundance of *Finegoldia*, *Dialister*, *Clostridium*, and *Eubacterium*. Infants on antibiotics at the time of sampling had lower relative abundance of *Clostridiaceae 02d06* and *Clostridium*, but higher relative abundance of *Trabulsiella*. There were no significant associations between bacterial genera in the fecal microbiota and group, time, or other covariates.

Associations between microbiota diversity and skin health measures including pH, TEWL, and NSCS were explored. Diversity of the microbiota was negatively correlated with skin pH, but no consistent relationships were observed between microbiota diversity and TEWL (S5 Fig). Diversity of the diapered skin microbiota was lower in infants with elevated neonatal skin condition scores (i.e. NSCS >3; n = 109 samples) than infants with normal NSCS (n = 414 samples; 3.98 vs. 4.14, p = 0.04). No clear relationships were observed between skin pH, TEWL, and NSCS and skin microbiota community structure (S6 Fig).

## Discussion/Conclusion

In this study, we sought to determine the impact of diaper change frequency and other clinical factors on the skin microbiota of preterm infants. We found that the diapered skin microbiota undergoes changes over time including increased alpha-diversity and shifts in the relative abundances of multiple taxa. Of the variables considered in our analyses, body site and interindividual variation were the primary factors associated with variation in microbiota composition and diversity. Reduced diaper change frequency did not impact diversity of the diapered skin microbiota but was associated with variation in microbiota community structure and relative abundances of certain bacterial genera. Infant diet and antibiotic exposures also contributed to variation in the diapered skin microbiota. These findings suggest that multiple factors including infant maturation, diapered skin care practices, and other treatments influence the development of the skin microbiota in preterm infants.

Prior studies have described that the microbiota is poorly differentiated across body sites in the early postnatal period, but sites rapidly progress to become more compositionally distinct in early infancy [5,26–30]. Most of the infants in our study were over one month old at time the intervention was initiated and differences between sites were already apparent. The skin chest microbiota was dominated by taxa typical of the skin microbiota, including *Staphylococcus* and *Streptococcus*, while the enrichment of gut-associated taxa in the buttocks skin microbiota, including *Enterobacteriaceae*, *Clostridium*, and strictly anaerobic taxa, is likely related to the occluded environment and contamination of the diapered skin with stool. Interindividual differences in microbiota community structure between samples were also readily apparent. Clinical covariates, including infant diet, delivery mode, and antibiotic exposures were associated with minor variations in microbiota composition, but not alpha-diversity. Consistent with other studies, most of the variation in the skin microbiota between samples was not attributable to any of the measured clinical variables [5,28]. It is possible that earlier initiation of the intervention and sampling may have revealed different effects of diaper change frequency and other covariates on development of the microbiota. We did not observe significant associations between clinical covariates and the fecal microbiota, though it should be noted that fewer fecal samples were collected than skin samples in this cohort.

The NSCS is a measure of skin condition based on erythema, dryness, and skin breakdown [32]. We found that diversity of the skin microbiota was lower among infants with an elevated NSCS, possibly indicating that a diverse microbiota is important for maintaining skin integrity in preterm infants. We also explored associations between skin pH and TEWL with microbiota diversity. While acidification of the skin may inhibit the growth of some organisms, we observed a weak negative correlation between alpha-diversity of the microbiota and skin pH. TEWL did not appear to have significant effects on microbiota diversity or composition.

A limitation of our study was that we used 16S rRNA gene sequencing, which lacks resolution to discriminate between strains and does not provide transcriptional data to demonstrate differences in microbial activity between groups. It is possible that diaper change frequency or other covariates considered in the models were associated with variations in specific strains or community functions that were not detected in our analyses. Another limitation is the lack of a priori sample size calculations for the microbiome-related outcomes. Despite these limitations, we did not observe an increased burden of pathogenic taxa or altered bacterial diversity among infants treated with reduced diaper change frequency. We previously reported that reduced diaper change frequency did not have negative effects on skin health measures [19]. Together, these findings suggest that reducing diaper change frequency is a safe strategy to minimize stress in medically stable preterm infants. It should be noted that these findings may not be generalizable to younger preterm infants in the first postnatal weeks, when skin barrier function is less mature.

The cutaneous and intestinal microbiomes influence maturation of the infant’s immune system, and multiple studies have shown that perturbations of the microbiome in early life may have both immediate and lasting impacts on immune development [31–33]. Infants born preterm are at increased risk for infection due to immature skin barrier and immune function, yet the impact of the skin microbiome on barrier maturation and the risk of infection remain poorly understood [34]. Future studies should continue to investigate how skin care practices in the NICU influence the development of the skin microbiome and skin barrier function to optimize outcomes in this vulnerable population. The study is registered in the Clinicaltrials.gov registry # NCT0337075.

## Supporting information

S1 Fig Rarefaction curves for all samples based on observed ASVs. (TIF)

S2 Fig Microbiota composition across body sites. Redundancy analysis (RDA) showing factors associated with variation in microbiota composition between samples. The overall RDA model explained 49.7% of the variance in microbiota composition (p<0.001). All factors included in the model (diaper change frequency, site, subject, observation week, delivery mode, number of antibiotic courses, postmenstrual age, and human milk diet) contributed significantly to the model (p<0.001). Explanatory variables are represented by blue arrows. Individual samples are represented by circles and color-coded according to site (orange for stool, yellow for skin buttocks, and green for skin chest). Red crosses represent individual microbial taxa. Sample sizes were n = 520 for chest, n = 528 for buttocks, and n = 131 for stool.
(TIF)

S3 Fig Average taxonomic composition at the end of the bundled care intervention (observation week 6) with OTUs grouped at the genus level. Composition was similar between bundled care groups. Only the top 43 most abundant genera are shown in the figure legend.
(TIF)

S4 Fig Redundancy analysis (RDA) model of skin buttocks samples. A subset of samples are color-coded according to individual subject to demonstrate distinct clustering within subjects regardless of diaper change frequency, observation week, or other factors.
(TIF)

S5 Fig Correlations between skin microbiota diversity, pH, and transepidermal water loss (TEWL). Significant negative correlations were observed between Shannon Diversity Indices and skin pH, but no consistent relationships were observed between Shannon Diversity Indices and TEWL. Samples from infants treated with every 3-hour diaper changes are shown in blue and samples from infants treated with every 6-hour diaper changes are shown in blue.
(TIF)

S6 Fig Relationship of skin buttocks microbiota composition with skin health measures. Non-metric Multidimensional Scaling (NMDS) plot of skin buttocks samples based on Bray-Curtis distances. There was no apparent clustering of samples according to skin pH, TEWL, or NSCS.
(TIF)

S1 Table Reporting checklist for randomized trial. Details of the location of data in the manuscript that follows the CONSORT guidelines for reports of a randomized trial.
(DOCX)

S2 Table Redundancy analysis of the microbiota across body sites. Redundancy analysis from all sites were included in an RDA model. Variables considered in the model included site (buttocks, chest, or stool), bundled care group, time on study (week), delivery mode (vaginal or cesarean delivery), subject, number of antibiotic courses, PMA, and diet (human milk). The resulting model explained 49.8% of the variation in microbiota community structure between samples. Of all variables considered in the model, site (buttocks, chest, or stool) accounted for most of the variation between samples (30.0%, p<0.001), followed by individual subject (6.1%, p<0.001), and delivery mode (3.3%, p<0.001).
(DOCX)
