# Supplementary figures and images for: The skin microbiota of preterm infants and impact of diaper change frequency
Source: PLoS One. 2024 Aug 1;19(8):e0306333. doi: 10.1371/journal.pone.0306333 (PMC11293746; doi:10.1371/journal.pone.0306333)

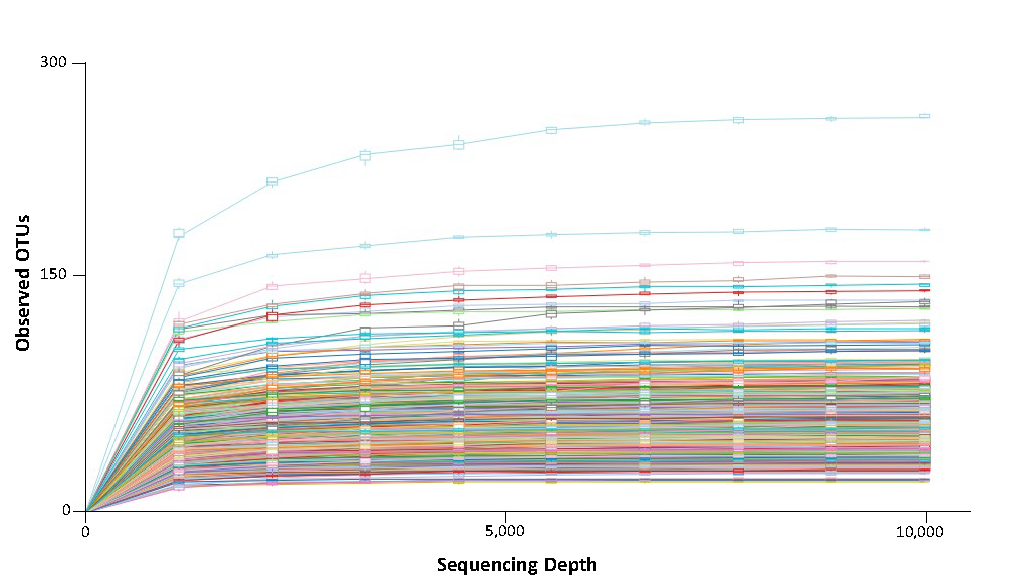

Supplement: S1 Fig — (TIF) [file pone.0306333.s001.tif]

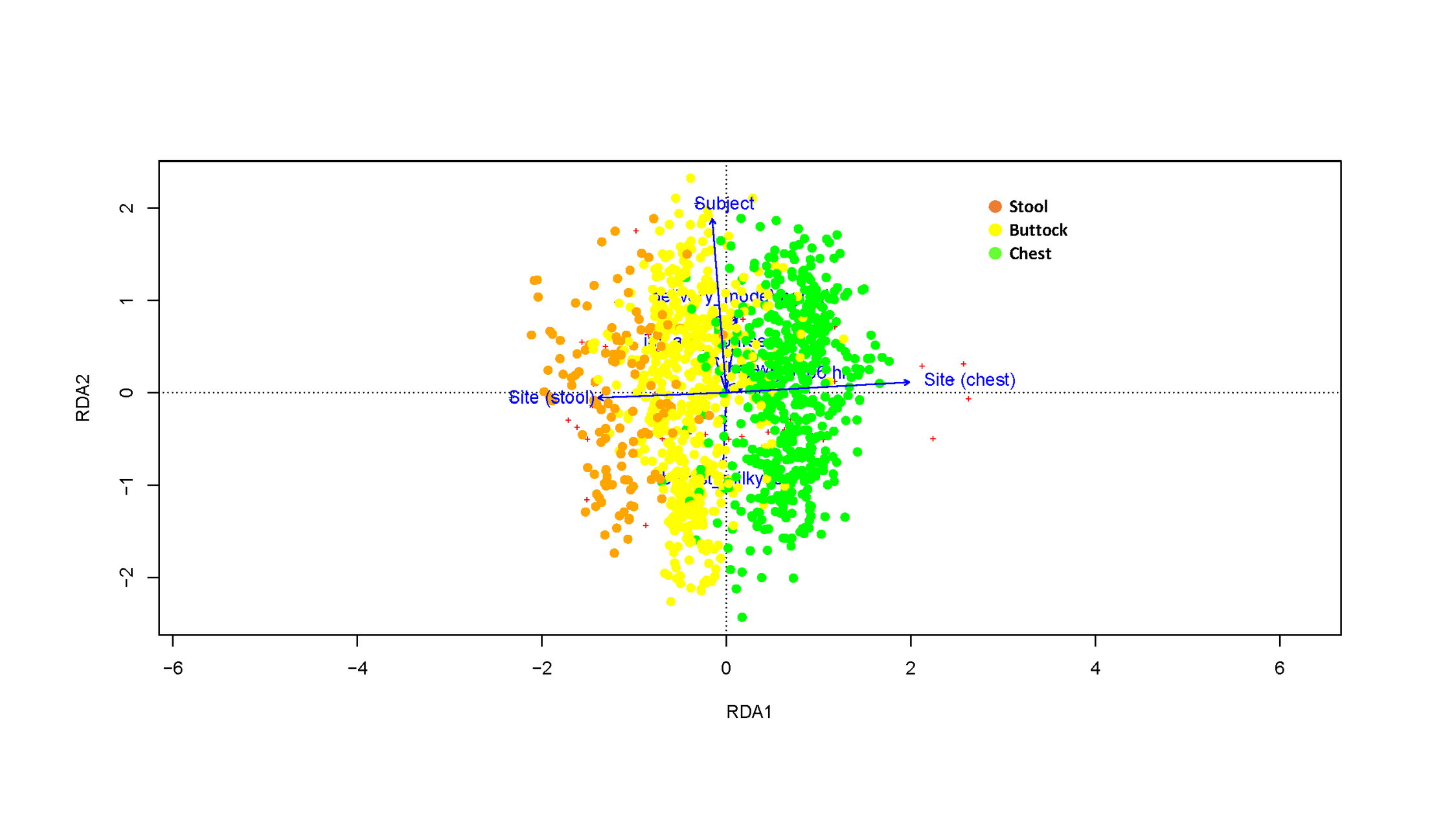

Supplement: S2 Fig — Redundancy analysis (RDA) showing factors associated with variation in microbiota composition between samples. The overall RDA model explained 49.7% of the variance in microbiota composition (p<0.001). All factors included in the model (diaper change frequency, site, subject, observation week, delivery mode, number of antibiotic courses, postmenstrual age, and human milk diet) contributed significantly to the model (p<0.001). Explanatory variables are represented by blue arrows. Individual samples are represented by circles and color-coded according to site (orange for stool, yellow for skin buttocks, and green for skin chest). Red crosses represent individual microbial taxa. Sample sizes were n = 520 for chest, n = 528 for buttocks, and n = 131 for stool. (TIF) [file pone.0306333.s002.tif]

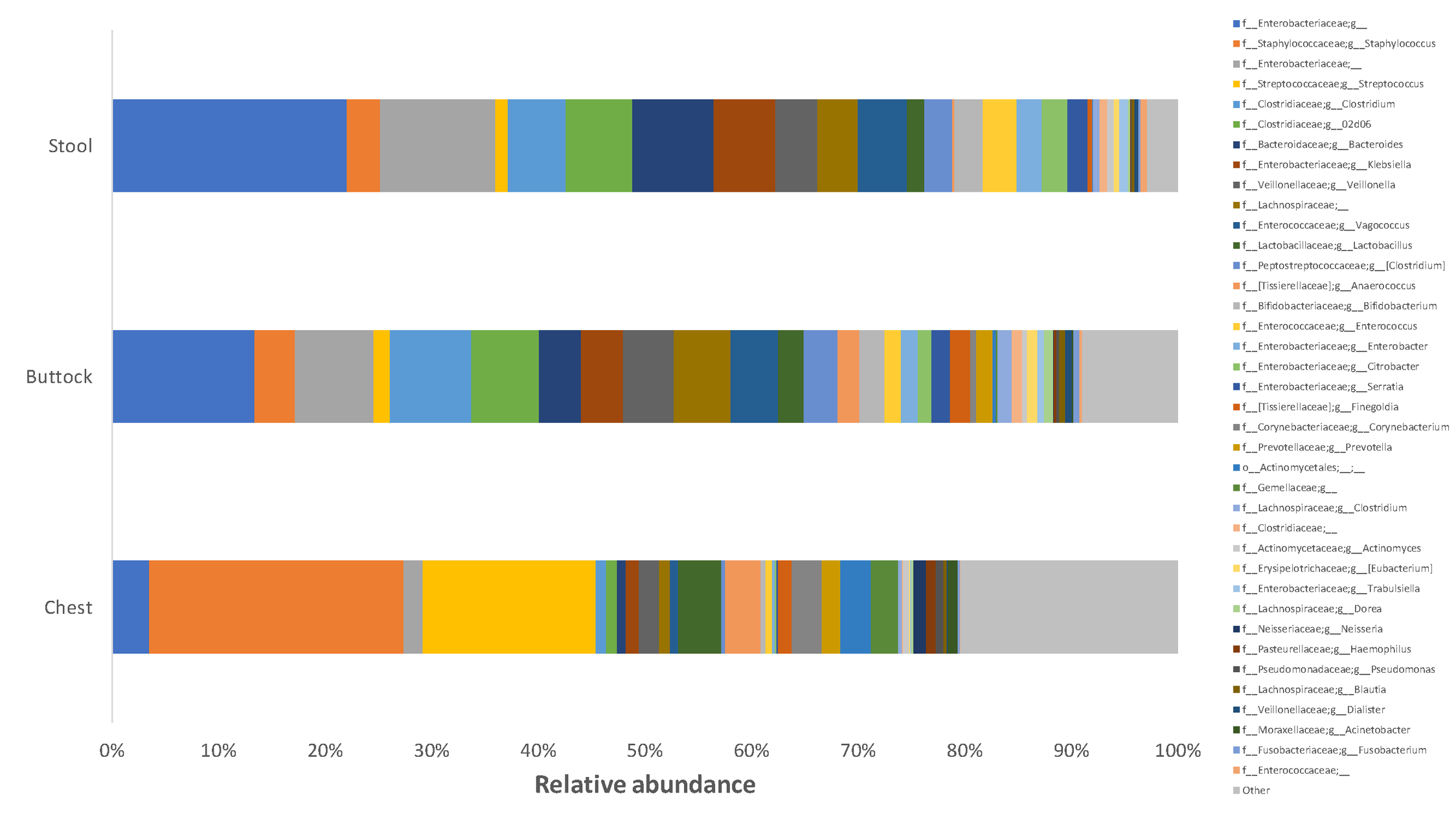

Supplement: S3 Fig — Composition was similar between bundled care groups. Only the top 43 most abundant genera are shown in the figure legend. (TIF) [file pone.0306333.s003.tif]

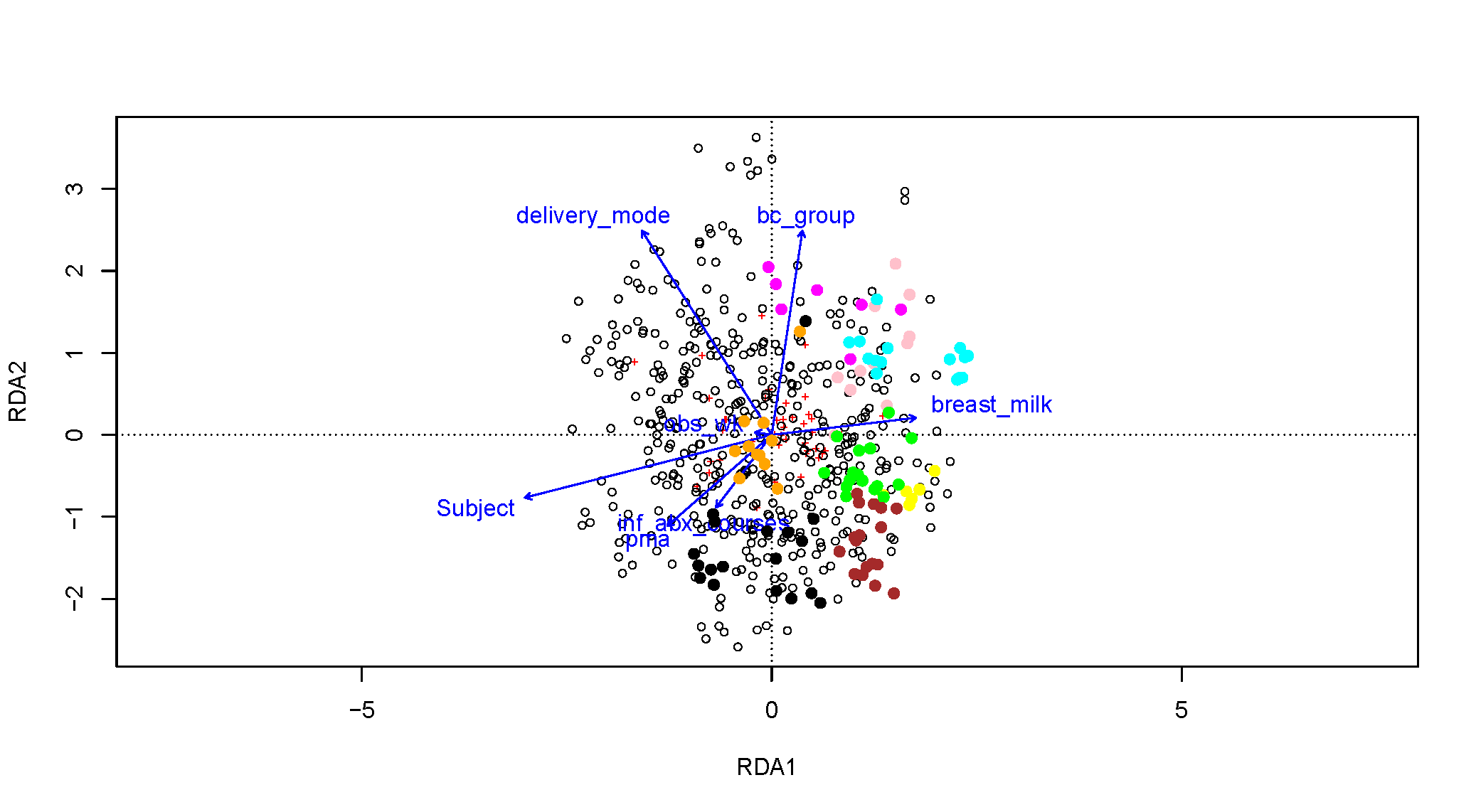

Supplement: S4 Fig — A subset of samples are color-coded according to individual subject to demonstrate distinct clustering within subjects regardless of diaper change frequency, observation week, or other factors. (TIF) [file pone.0306333.s004.tif]

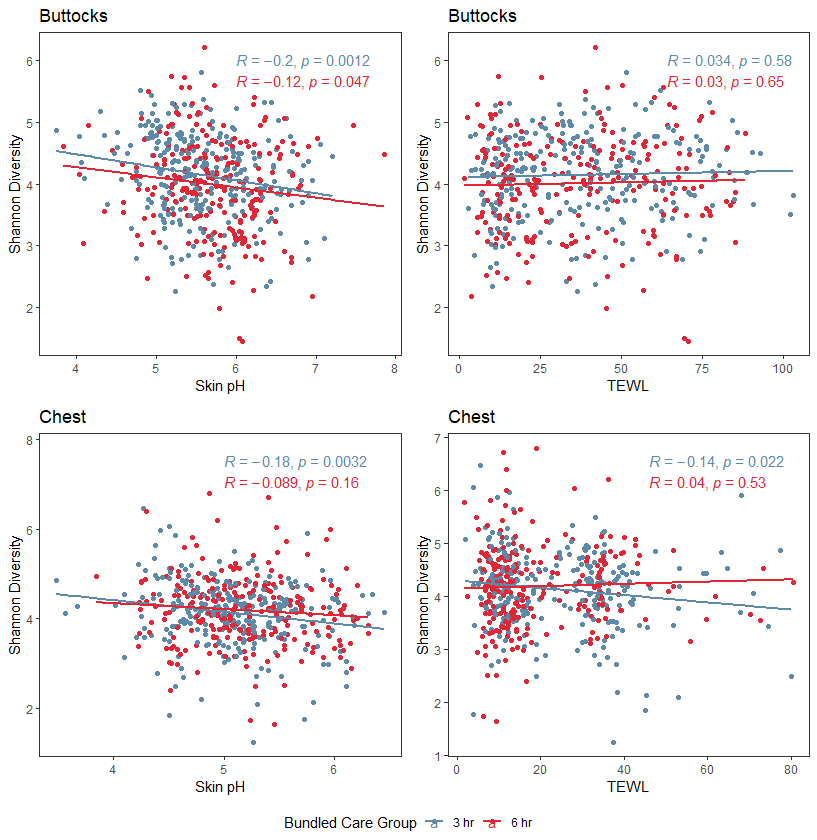

Supplement: S5 Fig — Significant negative correlations were observed between Shannon Diversity Indices and skin pH, but no consistent relationships were observed between Shannon Diversity Indices and TEWL. Samples from infants treated with every 3-hour diaper changes are shown in blue and samples from infants treated with every 6-hour diaper changes are shown in blue. (TIF) [file pone.0306333.s005.tif]

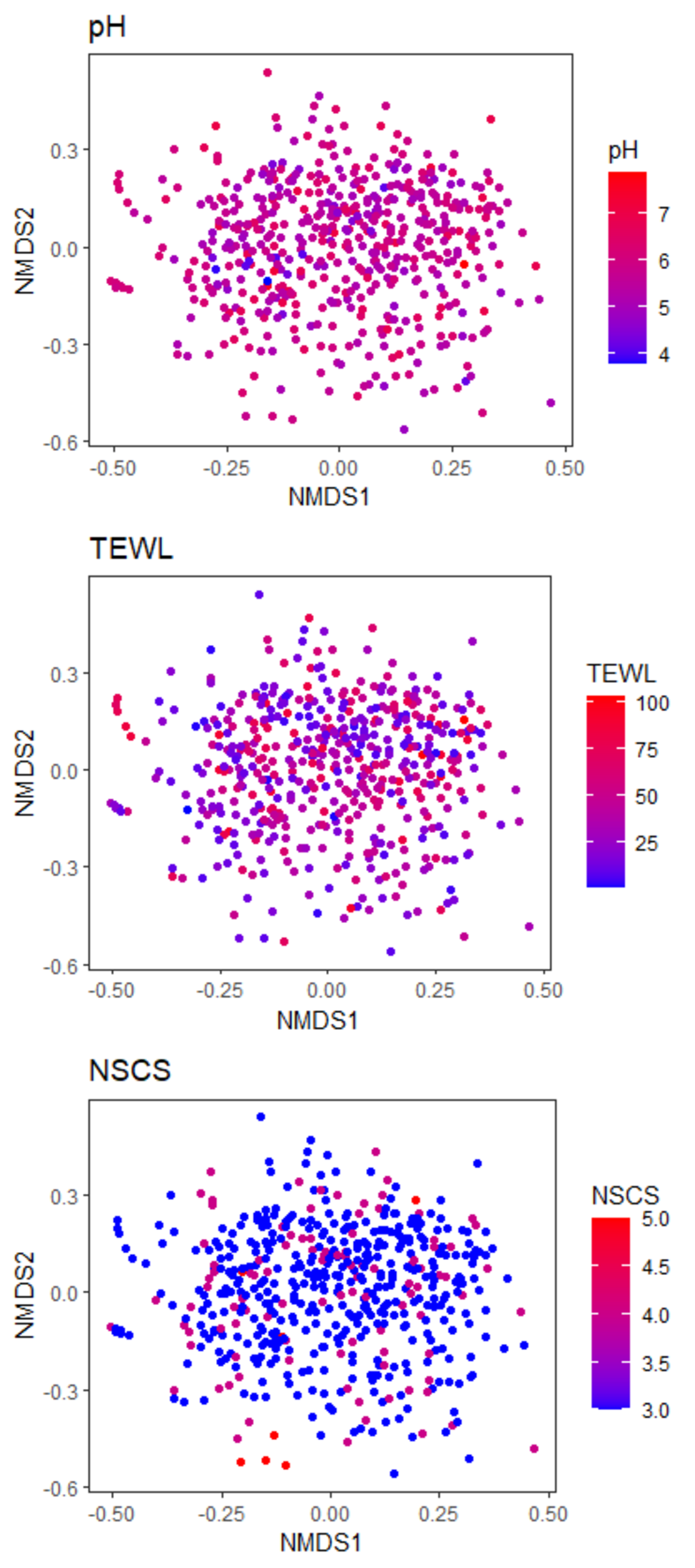

Supplement: S6 Fig — Non-metric Multidimensional Scaling (NMDS) plot of skin buttocks samples based on Bray-Curtis distances. There was no apparent clustering of samples according to skin pH, TEWL, or NSCS. (TIF) [file pone.0306333.s006.tif]
