# Supplementary material for: The skin microbiota of preterm infants and impact of diaper change frequency
Source: PLoS One. 2024 Aug 1;19(8):e0306333. doi: 10.1371/journal.pone.0306333 (PMC11293746; doi:10.1371/journal.pone.0306333)
Supplement: S2 Table — Redundancy analysis from all sites were included in an RDA model. Variables considered in the model included site (buttocks, chest, or stool), bundled care group, time on study (week), delivery mode (vaginal or cesarean delivery), subject, number of antibiotic courses, PMA, and diet (human milk). The resulting model explained 49.8% of the variation in microbiota community structure between samples. Of all variables considered in the model, site (buttocks, chest, or stool) accounted for most of the variation between samples (30.0%, p<0.001), followed by individual subject (6.1%, p<0.001), and delivery mode (3.3%, p<0.001). (DOCX) [file pone.0306333.s008.docx]

**S1 Table**. **Redundancy analysis of the microbiota across body sites.**

|  | **Df** | **Variance** | **F** | **Pr(>F)** |
| --- | --- | --- | --- | --- |
| Site | 2 | 30.037 | 97.2731 | 0.001 *** |
| Subject | 1 | 6.099 | 39.5013 | 0.001 *** |
| Delivery mode | 1 | 3.336 | 21.6086 | 0.001 *** |
| Diaper Change Group | 1 | 3.08 | 19.9492 | 0.001 *** |
| Antibiotic courses | 1 | 2.566 | 16.6181 | 0.001 *** |
| Postmenstrual age | 1 | 2.176 | 14.0935 | 0.001 *** |
| Breast milk feeding | 1 | 1.441 | 9.3329 | 0.001 *** |
| Observation week | 1 | 1.033 | 6.6907 | 0.001 *** |
